# Immunological effects of radiopharmaceutical therapy

**DOI:** 10.3389/fnume.2024.1331364

**Published:** 2024-04-04

**Authors:** Amanda G. Shea, Malick Bio Idrissou, Ana Isabel Torres, Tessa Chen, Reiner Hernandez, Zachary S. Morris, Quaovi H. Sodji

**Affiliations:** ^1^ Department of Human Oncology, University of Wisconsin School of Medicine and Public Health, Madison, WI, United States; ^2^ Department of Medical Physics, University of Wisconsin School of Medicine and Public Health, Madison, WI, United States; ^3^ Carbone Cancer Center, University of Wisconsin-Madison, Madison, WI, United States

**Keywords:** radiopharmaceutical therapy, radiation therapy, radionuclide, alpha-particle emitter, beta-particle emitter, auger electron emitter, metastatic cancer, immune system

## Abstract

Radiation therapy (RT) is a pillar of cancer therapy used by more than half of all cancer patients. Clinically, RT is mostly delivered as external beam radiation therapy (EBRT). However, the scope of EBRT is limited in the metastatic setting, where all sites of disease need to be irradiated. Such a limitation is attributed to radiation-induced toxicities, for example on bone marrow and hematologic toxicities, resulting from a large EBRT field. Radiopharmaceutical therapy (RPT) has emerged as an alternative to EBRT for the irradiation of all sites of metastatic disease. While RPT can reduce tumor burden, it can also impact the immune system and anti-tumor immunity. Understanding these effects is crucial for predicting and managing treatment-related hematological toxicities and optimizing their integration with other therapeutic modalities, such as immunotherapies. Here, we review the immunomodulatory effects of α- and β-particle emitter-based RPT on various immune cell lines, such as CD8+ and CD4+ T cells, natural killer (NK) cells, and regulatory T (Treg) cells. We briefly discuss Auger electron-emitter (AEE)-based RPT, and finally, we highlight the combination of RPT with immune checkpoint inhibitors, which may offer potential therapeutic synergies for patients with metastatic cancers.

## 1 Introduction

The intricate interplay between radiation-induced DNA damage and immune response underscores the evolving understanding of the impact of RT on tumor control. Historically, radiation-induced DNA damage has been regarded as the primary mechanism by which most solid tumors respond to ionizing radiation. This damage leads to various cellular responses, such as apoptosis, senescence, and autophagy, ultimately resulting in tumor control (1–3). Since these cytotoxic effects can also have deleterious effects on bone marrow and systemic immune cell populations, RT has been considered immunosuppressive (4, 5). Yet, emerging data suggests that RT has the potential to elicit a favorable immune response by stimulating the immune system, which in turn contributes to tumor eradication (6–8). However, harnessing these therapeutic benefits hinges on preserving the function of effector immune cells amidst the deleterious effects of radiation (8–10). Recent advances in diagnostic imaging, tumor delineation, and motion management have resulted in the accurate delivery of radiation to the tumor while sparing healthy organs and minimizing hematological toxicities, including immune cell depletion (11). Despite these advances, EBRT cannot effectively be used to irradiate all disease sites in patients with widespread metastatic disease due to radiation-induced toxicity, including bone marrow and hematologic toxicities (12). In contrast to EBRT, RPT combines a radionuclide (radioactive isotope) with a tumor-targeting agent (e.g., antibody, peptide, small molecule) that can potentially deliver systemic radiation to all tumor sites. Although RPT shows considerable potential in the oncology field, it faces numerous limitations, including the restricted availability of certain radionuclides and biodistribution (13–15). Additionally, RPT can also have dose-limiting toxicities. In this review, we will cover the two most common groups of therapeutic radionuclides: alpha (α) and beta (β−) emitters. While the effects of EBRT on the immune system and healthy tissues are beginning to be understood and are still being explored, we are only starting to understand the effects of RPT on the various immune cell lineages. Here, we summarize existing pre-clinical and clinical data describing the impact of RPT on CD8+ T cells, CD4+ T cells, NK cells, and Treg cells.

## 2 Immunomodulatory effects of radiation therapy

Clinically, RT is delivered by EBRT or brachytherapy, with EBRT representing the more commonly used treatment modality (16–18). EBRT is a mainstay of oncological care and has been a pillar of cancer therapy for more than a century (19). As such, the majority of the described immunomodulatory effects of RT have been gleaned from radiation delivered by EBRT (6, 20, 21). In addition to inducing lethal DNA damage resulting in cell death, RT can induce immunogenic cell death (ICD) of tumor cells, which is characterized by the extracellular release of adenosine triphosphate (ATP) and high-mobility group box 1 protein (HMGB1) (22–25). RT can also induce calreticulin translocation from the endoplasmic reticulum onto the cell surface, which can result in immune cell recognition, infiltration in the tumor microenvironment (TME), and activation (26–30). RT-induced ICD can stimulate antigen presentation by dendritic cells (DCs), activating cytotoxic effector cells like CD8+ T cells (31). Additionally, RT sensitizes tumor cells to immune-cell-mediated killing by upregulating MHC-1 expression and inducing the release of pro-inflammatory cytokines via a type 1 interferon (IFN) response, which further stimulates CD8+ T cells (7, 32–36). While the immunomodulatory effects of RT delivered by EBRT are being elucidated, RT can also be detrimental to healthy cells, including immune cells (12, 37, 38). These effects are of increased concern with large radiation fields, such as those that would be needed to treat all lesions in patients with widely metastatic cancer.

## 3 Limitations of RT delivered as EBRT

**Figure 1:**
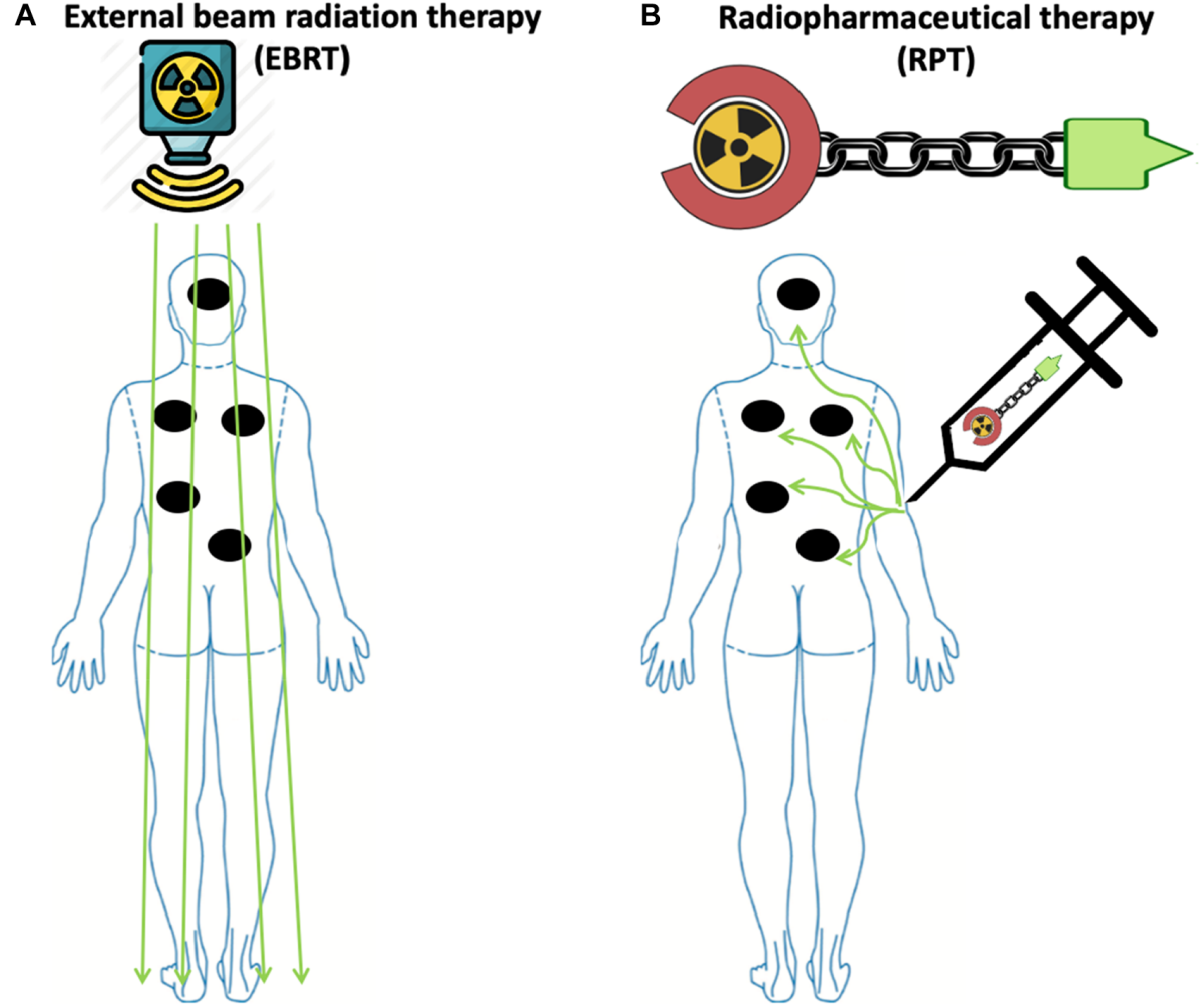
Radiopharmaceutical therapy delivers radiation to all sites of disease with potentially less toxicity compared to external beam radiation therapy in widely metastatic disease. (**A**) To irradiate all sites of disease in patients with widely metastatic disease, including microscopic disease using EBRT, a large radiation field is needed, thus increasing the risk of toxicity. (**B**) In contrast to EBRT, due to the molecular targeting in RPT, radiation is delivered to malignant cells expressing the target, while irradiation to healthy tissue is minimized due to the differential expression of the target. Made with Biorender.com.

EBRT can cause potentially harmful side effects on the healthy tissue surrounding the tumor (11, 18, 39). In addition, lymphopenia is a concerning side effect of radiation observed in several solid tumors, such as high-grade glioma, head and neck, lung, esophageal, pancreatic, and cervical cancers treated with EBRT (40–48). The observed lymphopenia appears to correlate with not only the size of the radiation field, but also the radiation dose delivered to lymphoid organs such as the spleen and lymph nodes (49, 50). Moreover, exposure to therapeutic radiation can lead to the development of secondary malignancies (51). The risk of such secondary malignancies is also associated with the size of the radiation field (52). Furthermore, EBRT cannot effectively target radiographically occult lesions. Thus, it is generally not used when there is a need to irradiate all tumor sites in patients with metastatic cancers (Figure 1). In these instances, RPT is an attractive therapeutic option that can deliver radiation systematically to all metastatic lesions (15, 53).

## 4 Radiopharmaceutical therapy: an alternative to EBRT in metastatic disease

**Figure 2:**
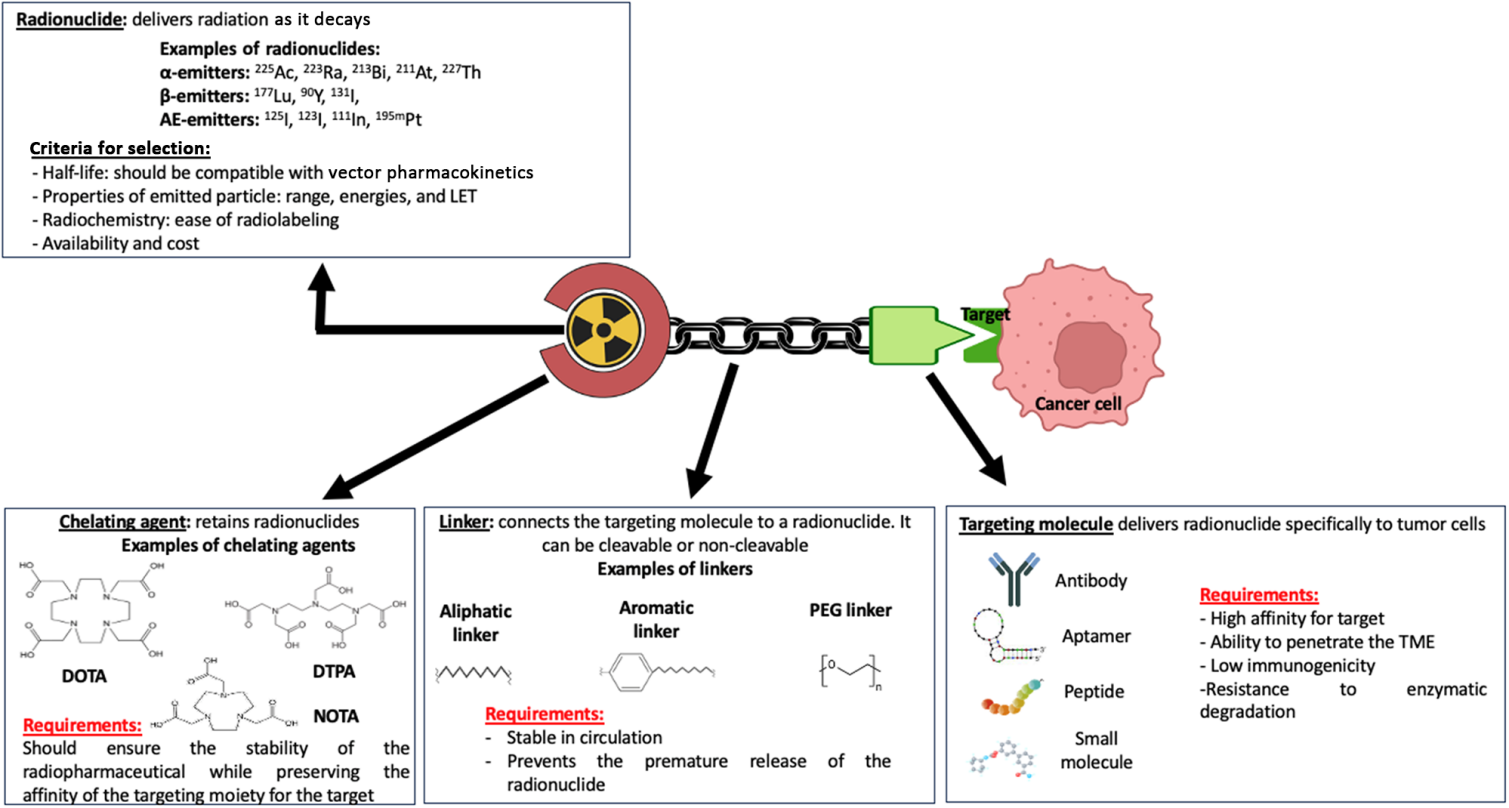
Pharmacophoric model of a radiopharmaceutical agent. The therapeutic effect of a radiopharmaceutical is impacted by the properties of the various domains of the pharmacophoric model, including the radionuclide, chelating agent, linker, and targeting molecule or moiety. Made with Biorender.com.

RPT is a growing class of cancer therapeutics in which a radionuclide is linked to a ligand such as a small molecule, peptide, or antibody directed toward a cell surface antigen upregulated on malignant cells (15, 54) (Figure 2). Following the intravenous administration of a radiopharmaceutical agent, it selectively accumulates in the tumor and the TME, thus sparing healthy tissues that do not express the targeted antigens and allowing the targeted delivery of radiation to malignant cells. This makes RPT particularly attractive for the treatment of metastatic and microscopic tumors (15, 55–57). Indeed, several radiopharmaceutical agents have been shown to increase the survival of patients with metastatic diseases, for example prostate cancer and neuroendocrine cancer (58–60).

The recent approvals by the FDA of ^223^Ra-dichloride (^223^RaCl_2_; Xofigo®) for the treatment of castration-resistant prostate cancer with symptomatic bone metastases, ^177^Lu-DOTATATE (Lutathera®) for gastroenteropancreatic neuroendocrine tumors (GEP-NET), and ^177^Lu-PSMA-167 (Pluvicto®) for metastatic castration-resistant prostate cancer (mCRPC) have resulted in a renewed enthusiasm for the development of novel RPT agents (61–63).

Radionuclides used for RPT can emit different forms of therapeutic radiation, for example α-particles, β-particles, γ-rays, and Auger electrons. Often, a given radionuclide will emit multiple forms of radiation before decaying to a non-radioactive element. Each form of emitted radiation is characterized by unique properties, including range in biological tissues, relative biological effectiveness (RBE), physical half-life, and linear energy transfer (LET), which is the energy released per unit of distance (54, 55, 64). While RPT offers the advantage of targeted radiation delivery to tumor cells, it can induce dose-limiting toxicities including bone marrow and hematologic toxicity, nephrotoxicity, neurotoxicity, and hepatotoxicity (65–69).

β-particle emitters, which include Lutetium-177 (^177^Lu), Yttrium-90 (^90^Y), and Iodine-131 (^131^I) are currently the most commonly used radionuclides employed in RPT. β-particles have a low LET (∼0.2 keV/µm), and their range in biological tissue is several millimeters (up to 12 mm) (54, 70), making them well-suited for the irradiation of large tumors and tumors with heterogeneous expression of the RPT antigen target. Due to their low LET, β-emitters induce single-stranded DNA breaks, DNA base modifications, and DNA-protein crosslinks, which are more readily repairable compared to double-stranded DNA breaks (71, 72).

α-particle emitters include radionuclides such as Radium-223 (^223^Ra), Actinium-225 (^225^Ac), and Astatine-211 (^211^At). They have a short tissue range (50–100 µm), making them suitable for the treatment of micrometastases, and a high LET (50–230 keV/µm), thus making them highly cytotoxic (54, 71, 73). α-particles mostly induce clusters of DNA damage, including double-stranded DNA breaks, which are difficult to repair (54). The relative biological effectiveness (RBE) for α-particles is 5-fold that of β particles, highlighting the higher therapeutic potential of α-emitters compared to β-emitters (74).

Auger electron emitters include radionuclides such as Iodine-123 (^123^I), Iodine-125 (^125^I), and Indium-111 (^111^In). While they have a very short range in tissue (< 1 µm), they possess a medium to high LET (4–26 keV/µm) (54, 57, 73). Because of the short range, therapeutic benefits may be achieved with AEEs when they are in proximity of a sensitive cellular target such as nuclear DNA.

The majority of β-particle emitters and AEEs can also emit γ rays as they decay, which can be used for imaging purposes (75). The utilization of γ rays for imaging to assess the distribution of the uptake RPT is crucial for verifying that the uptake pattern aligns with the intended therapeutic target and for estimating the absorbed doses in both the target tissue and organs at risk. For example, γ rays emitted by ^177^Lu and ^131^I can be used to monitor *in vivo* biodistribution (76, 77). In some instances, data subsequently gleaned from such biodistribution can be used to increase the radiation dose and enhance therapeutic benefits (78, 79).

## 5 Immunomodulatory effects of RPT

**Figure 3:**
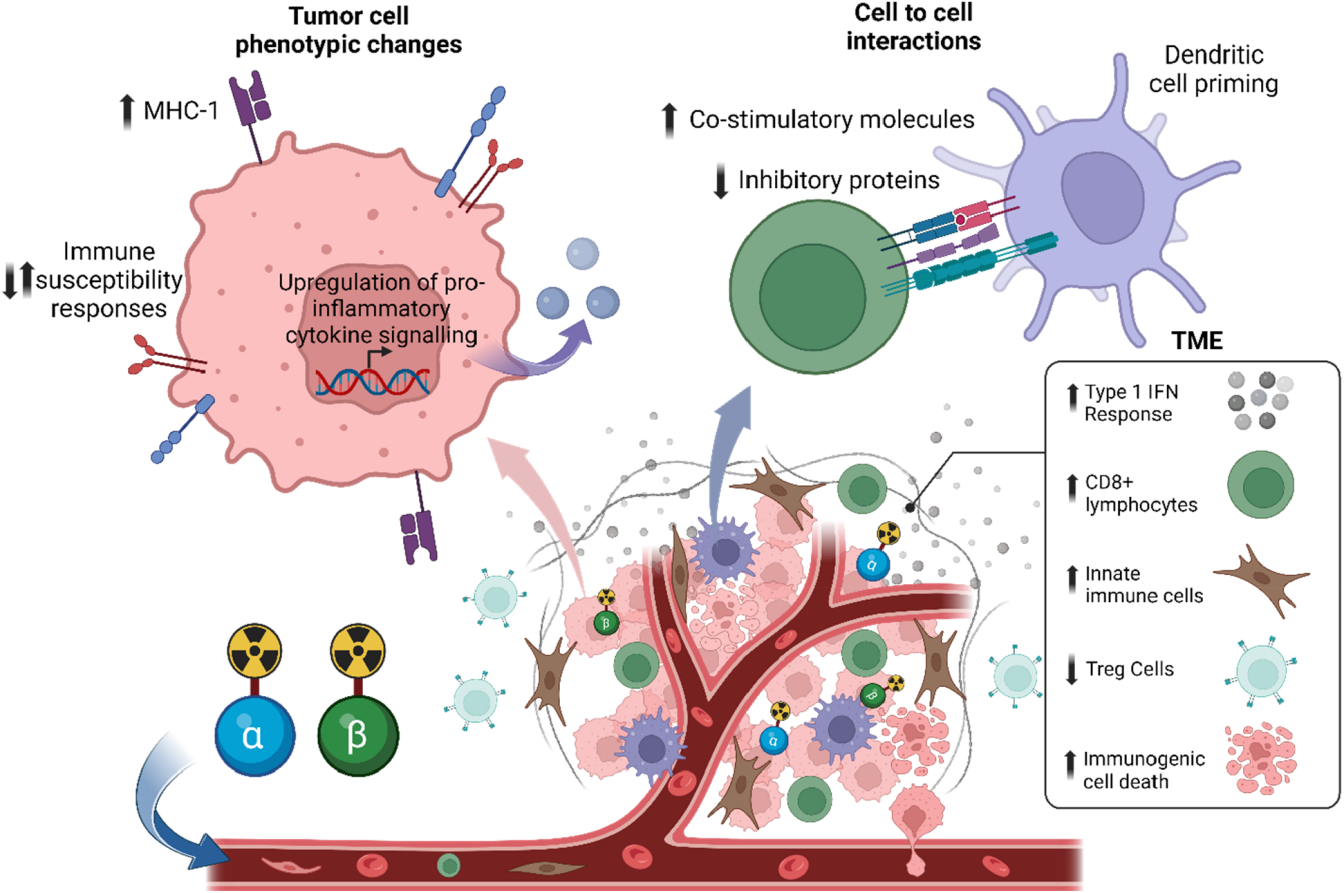
Systemic radiopharmaceutical treatment may have direct and indirect effects on the tumor microenvironment and immune cells. Preclinical data suggests that RPT with α-particles or β-particles has an immunostimulatory effect, such as an increase in type 1 interferon response, a decrease in regulatory T cells (Tregs), and an increase in immunogenic cell death.

Similar to EBRT, RPT may also influence the immune system's response to tumors (7, 8, 80–86). This can be achieved through the direct or indirect irradiation of immune cells in the TME (87) (Figure 3). The latter, known as the “bystander effect”, refers to the phenomenon where cells that are not directly exposed to radiation exhibit biological responses as a result of signals emitted by neighboring irradiated cells (88, 89). However, in the context of RPT, this classic definition has been challenged. Emerging data, such as those by Leung et al., suggest that bystander cells include irradiated and non-irradiated cells, while the bystander effect emanates from irradiated cells, and this contributes to the mechanism by which ^223^Ra is able to delay the development of metastatic lesions (90–92). Indeed, signaling molecules released from irradiated cells may not only affect non-irradiated adjacent cells, but may also affect irradiated cells in the vicinity. Moreover, the abscopal effect, defined as tumor regression outside the irradiated region, is also thought to be involved in the biological effects of RPT (87). However, because of the different physical properties of the radionuclides, the effects of RPT on immune cells may differ. Here, we provide a summary of relevant studies evaluating the immunomodulatory effects of α-emitter, β-emitter, and AEE-based RPT.

### 5.1 Effects of β-particle-emitter-based RPT on immune cells

#### 5.1.1 Preclinical studies

^177^Lu and ^131^I are examples of radionuclides that emit β-particles and γ rays, while ^90^Y is an example of an almost pure β-emitting radionuclides. With the recent FDA approval of ^177^Lu-based RPTs Lutathera® and Pluvicto®, there has been an increased and renewed interest in not only understanding the mechanism of action on the TME but also the hematological side effects of RPT and dose-dependent toxicities in the clinic (59, 60, 62, 63). Several studies have evaluated the safety and immunomodulatory effects of β-particle RPT in the preclinical setting (7, 8, 81, 93, 94). β-particle RPT can indirectly regulate the function of immune cells in the TME through anti-tumor response. For example, ^90^Y-NM600 (a radiolabeled tumor-targeting alkylphosphocholine analog) triggers a type 1 interferon response in irradiated malignant cells (7, 8). Furthermore, β-particle RPT can also induce ICD in tumor cells (15, 64). Rouanet and colleagues demonstrated that ^131^ICF01012 induced the release of damage-associated molecular patterns (DAMPs) such as HMGB1 and ATP into the extracellular milieu. ^131^ICF01012 also induced cell surface translocation of calreticulin, along with the secretion of type I IFN (80). Here, we will discuss the direct effects of β-particle-emitter RPT on specific immune cell populations.

##### 5.1.1.1 Regulatory T (Treg) cells

Following administration of ^90^Y-based RPT, FOXP3+ immunohistochemistry (IHC) staining revealed a notable reduction of Treg cells in the TME of tumors generated by the EL4 lymphoma cell line in murine syngeneic models (81).

##### 5.1.1.2 CD4+ T cells

In murine models of medullary breast adenocarcinoma, Vito and colleagues observed that while the ^177^Lu-BSA-tetrazine RPT did not significantly impact the level of CD4+ T cells in the peripheral blood after 4 days, an analysis of the TME by IHC demonstrated a significant decrease in intratumoral CD4+ T cells (95). Notably, Vito et al. did not analyze the CD4+ T cell subset to determine if they were specifically Tregs, however, Tregs are defined as CD4+/Foxp3+, indicating that this treatment combination effectively diminished nearly all CD4+ T cells. Similar to the observations of Hernandez et al., the findings by Vito et al. suggest that this treatment reduces the immunosuppressive tumor environment. In addition to this direct effect of ^177^Lu RPT reported by Vito et al., another study highlighted the indirect effect of ^90^Y-NM600 RPT on CD4+ T cells. Jagodinsky and colleagues co-cultured murine B16 melanoma or MOC2 head and neck squamous cell carcinoma cells in ^90^Y-NM600 until the tumor cells received a cumulative radiation dose of 6 Gy. They found that the viability of CD4+ T cells in the presence of ^90^Y-NM600 was enhanced when co-cultured with previously irradiated MOC2 and B16 tumor cells. Whereas in the absence of tumor cells, irradiation of CD4+ T cells with ^90^Y-NM600 significantly reduced their viability. They also investigated the expression of the immune checkpoint marker CTLA-4 and the immune activation signal IFN-γ on the T cell surface. They found that CTLA-4 was significantly upregulated on the CD4+ T cells in the presence of ^90^Y-NM600-treated MOC2 or ^90^Y-NM600-treated B16 cells, compared to the untreated controls. Additionally, they found that in the same experiment, IFN-γ was not significantly changed (7). This suggests that RPT to tumor cells induces signaling to CD4+ T cells that increases viability, but also increases immune inhibitory signals, potentially indicating a need for immunotherapy to block the upregulated immune checkpoint pathways.

##### 5.1.1.3 CD8+ T cells

Following treatment with ^90^Y-NM600 RPT, mice bearing EL4 lymphoma tumors revealed not only an increased infiltration of CD8+ T cells in the TME but also a significant activation of these infiltrated CD8+ T cells (81). Conversely, in a murine model of medullary breast adenocarcinoma, intratumorally delivered ^177^Lu-BSA-tetrazine RPT significantly decreased CD8+ T cells in the TME; however, this decrease was not seen in the CD8+ T cells in the peripheral blood (95). This was likely due to the targeting agent that was used, as in another experiment they found that intravenous administration of ^177^Lu-BSA-tetrazine RPT had poor tumor retention and high uptake in other tissues. Intratumoral delivery of ^177^Lu-BSA-tetrazine RPT allowed for local treatment while minimizing the systemic effect on peripheral CD8+ T cells. As described for CD4+ T cells, co-culture of CD8+ T cells with MOC2 or B16 tumor cells previously irradiated with ^90^Y-NM600 RPT indirectly enhanced the proliferation and activation of CD8+ T cells as demonstrated by increased viability and an increased secretion of IFN-γ. Moreover, CTLA-4 was similarly upregulated on the CD8+ T cells (7). In a murine model of prostate cancer, Potluri et al. observed that ^90^Y-NM600 RPT increased the infiltration of CD8+ T cells in the TME. However, similar to the observations by Jagodinsky et al., immune checkpoints including PD-1, CTLA-4, LAG-3, and VISTA were upregulated on the CD8+ T cells. They also revealed that the CD8+ T cell compartment showed an early increase in effector memory CD8+ T cells, with no increase in either the central memory, resident memory, or short-lived effector cells (93). While Hernandez et al. did not quantify specifically the memory populations following ^90^Y-NM600 treatment of EL4 tumors, they found in a rechallenge experiment that previously treated complete responder mice inoculated again with EL4 did not grow tumors, suggesting that RPT can induce memory formation (81).

##### 5.1.1.4 Natural killer (NK) cells

Patel et al. found that NK cell numbers were significantly increased in the TME of B78 tumors, a murine melanoma model, following ^90^Y-NM600 (8). A similar effect on NK cells was noted when a murine model of human neuroendocrine tumors was treated with ^177^Lu-based RPT. Additionally, such treatment enhances the activation of the status of infiltrated NK cells, as demonstrated by their expression of Fas ligand (FasL) (96).

##### 5.1.1.5 Dendritic cells and macrophages

Several preclinical studies have reported a decrease in macrophages in the TME following ^177^Lu-based RPT (81, 95). By staining for the macrophage F4/80 antigen, it was found that ^177^Lu-BSA-tetrazine RPT moderately decreased the infiltration of macrophages in the TME of medullary breast adenocarcinoma (95). Potluri et al. showed that 3 days after ^90^Y-based RPT, dendritic cells (DCs) from a murine prostate cancer model showed a non-significant increase in PD-L1 expression (93). Wu et al. demonstrated that ^177^Lu-DOTATATE increased the infiltration of antigen-presenting cells expressing CD86+ within the TME of a murine model of a human neuroendocrine tumor (96).

Through these preclinical studies, it has become clear that systemically delivered RPT has dynamic effects on the immune cells present in the TME. In several of the aforementioned studies, RPT monotherapy did not elicit a complete tumor response. As such, many included a combination with immune checkpoint inhibition (ICI) to synergize the immunomodulatory effects of the RPT (7, 8, 81, 93). This indicates a need for careful study of the immune cell types present in the TME, along with the activation and exhaustion status, to determine which, if any, tumor models will benefit from RPT monotherapy or require combinations with other therapies, such as immunotherapy, to increase the effectiveness of the RPT.

#### 5.1.2 Clinical studies

Peptide receptor radionuclide therapy (PRRT) has been clinically employed for nearly two decades for the management of selected malignancies (97). PRRT is safe and considered to be an effective treatment for two types of solid tumors: neuroendocrine tumors (NET) and gastroenteropancreatic neuroendocrine neoplasms (NENs). ^90^Y-octreotide and ^177^Lu-ocreotate are the two most common PRRTs used for NETs and NENs (98, 99). Clinically, these β-particle-emitter-based PRRT agents show that less than 13% of patients exhibit grade 3 or higher hematological toxicities. However, in rare cases, myelodysplastic syndromes or acute leukemia have been reported. Although the expected absorbed doses were below the conventional toxicity threshold, the persistence of acute and long-lasting bone marrow toxicities still raises concerns, especially with repeated administration of PRRT (97). Phase 1 studies have suggested that the upper limit of total activity per cycle of ^90^Y to minimize hematological toxicity is 5.18 GBq (98). The rate of hematological toxicity is further increased in patients who received cytotoxic chemotherapy prior to PRRT agents. To this end, Kwekkeboom et al. showed that the rate of grade 2 or 3 leukocytopenia or thrombocytopenia was 67% in patients with a history of chemotherapy prior to ^177^Lu-based PRRT compared to 22% in patients without prior chemotherapy (100).

Two seminal phase 3 trials led to the FDA approval of ^177^Lu-DOTATATE and ^177^Lu-PSMA-617. In the NETTER-1 trial, 116 patients were administered 7.4 GBq ^177^Lu-DOTATATE every 8 weeks for 4 cycles. In total, 9% of patients had a grade 3 or higher lymphopenia (60). In the phase 3 VISION trial, which enrolled 831 patients with mCRPC, patients received either standard of care only or standard of care plus ^177^Lu-PSMA-617. Compared to the standard of care-only group, there was a 38% reduction in the risk of death and a 60% decrease in disease progression with the addition of ^177^Lu-PSMA-617 to the standard of care. Although side effects were reported, these did not impact the quality of life of the patients (59). Further analysis of the VISION trial indicated that the average total absorbed dose for 6 × 7.4 GBq (44.4 GBq) was 1.5 Gy (±0.9) in the bone marrow (101). Moreover, in the cohort receiving ^177^Lu-PSMA-617, the rate of hematological toxicity correlated with the extent of bony metastatic disease burden, as the absorption in these metastatic sites resulted in the irradiation of adjacent bone marrow (102).

Clinically, the β-particle emitter radioisotopes have been shown to be well tolerated and safe. The renewed interest in the field has inspired new clinical trials and unique approaches to better target tumors. However, many of the clinical trials’ primary outcomes is overall survival or disease-free progression, with few analyzing the effects on the systemic immune system, only on the hematologic toxicities of the radionuclides. Understanding the effects and limits of each of these RPTs in the TME and on the immune cells present will be critical to determining optimal combinations of RPTs and immunotherapy, including ICI, in the future.

#### 5.1.3 Toxicities induced by β-particle-emitter-based RPT

Because damage induced by β-particle emitters may be easily repaired, higher doses may be necessary to achieve a therapeutic response, which can paradoxically lead to more treatment-related toxicities. Bone marrow and hematologic toxicity, such as anemia, leukopenia, and thrombocytopenia, are the frequent side effects of β-particle emitter-based RPT (103). Furthermore, some β-emitting radiopharmaceuticals are excreted through the kidneys, posing a risk of nephrotoxicity (104). We have summarized the major clinical toxicities observed during the phase 3 trials that led to FDA approval of ^177^Lu-PSMA-617 in mCRPC (Table 1) and of ^177^Lu-DOTATATE for metastatic midgut neuroendocrine tumors (Table 2).

**Table 1:** Summary of clinical toxicities of β-particle-emitter-based RPT: lutetium-117-PSMA-617 (59).

Radionuclide	Lutetium-117			
Targeting agent	PSMA-617			
Clinical indication	Metastatic castration-resistant prostate cancer			
Treatment arm	Standard of care alone		^177^Lu-PSMA-617 + standard of care	
Patients (%)	*N* = 205 (100%)		*N* = 529 (100%)	
Adverse event	All grades	Grade ≥3	All grades	Grade ≥3
Hematologic				
• Anemia	27 (13)	10 (5)	168 (32)	68 (13)
• Thrombocytopenia	9 (4)	2 (1)	91 (17)	42 (8)
• Neutropenia	NR	NR	NR	NR
• Lymphopenia	8 (4)	1 (0.5)	75 (14)	41 (8)
Non-hematologic				
• Constipation	23 (11)	1 (0.5)	107 (20)	6 (1)
• Diarrhea	6 (3)	1 (0.5)	100 (19)	4 (1)
• Nausea	34 (17)	1 (0.5)	187 (36)	7 (1)
• Vomiting	13 (6)	1 (0.5)	100 (19)	5 (1)
• Fatigue	47 (23)	3 (1.5)	228 (43)	31 (6)

NR, not reported.

**Table 2:** Summary of clinical toxicities of β-particle-emitter-based RPT: lutetium-117-dotatate (60).

Radionuclide	Lutetium-117			
Targeting agent	Dotatate			
Clinical indication	Metastatic midgut neuroendocrine tumors			
Treatment arm	Octreotide alone		^177^Lu-Dotatate + Octreotide	
Patients (%)	*N* = 110 (100%)		*N* = 111 (100%)	
Adverse event	All grades	Grade ≥3	All grades	Grade ≥3
Hematologic				
• Anemia	6 (5)	0 (0)	16 (14)	0 (0)
• Thrombocytopenia	1 (1)	0 (0)	28 (25)	2 (2)
• Neutropenia	6 (5)	1 (1)	6 (5)	1 (1)
• Lymphopenia	2 (2)	0 (0)	20 (18)	10 (9)
Non-hematologic				
• Constipation	NR	NR	NR	NR
• Diarrhea	21 (19)	2 (2)	32 (29)	3 (3)
• Nausea	13 (12)	2 (2)	65 (59)	4 (4)
• Vomiting	11 (10)	1 (1)	52 (47)	8 (7)
• Fatigue	28 (25)	2 (2)	44 (40)	2 (2)

NR, not reported.

### 5.2 Effect of α-particle-emitter-based RPT on immune cells

#### 5.2.1 Preclinical studies

The advantages of α-particles resulting from their physical properties (short tissue range and high LET) and the regulatory approval of ^223^RaCl_2_ for patients with mCRPC have stimulated the development of several α-particle-emitter-based RPTs. Several reports indicate that α-particle irradiation elicits immune activation (82, 84, 105). Here, we will discuss some preclinical studies that have evaluated the effects of α-particles on specific immune cell populations.

##### 5.2.1.1 Regulatory T (Treg) cells

Ferreira et al. showed that ^225^Ac-NM600 abrogated the infiltration of Treg cells in the TME of murine prostate cancer (105). In this study, they compared the anti-tumor and immune modulatory effects of ^225^Ac-NM600 and ^177^Lu-NM600 in two different immunocompetent prostate cancer models: MyC-Cap in FVB/NJ mice and Tramp-C1 in C57BL/6 mice. They injected these mice intravenously with either 7.4 or 18.5 kBq of ^225^Ac-NM600, or 5.5 or 18.5 MBq of ^177^Lu-NM600. After demonstrating the higher anti-tumor effect of ^225^Ac-NM600 compared to ^177^Lu-NM600, the immune response was compared. Immunophenotyping of the TME revealed that 7.4 or 18.5 kBq of ^225^Ac-NM600 resulted in a significant decrease of Tregs at 28 days post-injection in the Tramp-C1 TME and at 7-, 14-, and 28-days post-injection in the MyC-Cap TME, whereas ^177^Lu-NM600 had no effect. As Tregs are immunosuppressive cells, their decrease in the TME is beneficial for the anti-tumor response and could explain the superior anti-tumor effect observed with ^225^Ac-NM600 compared to ^177^Lu-NM600.

##### 5.2.1.2 CD8+ T cells

Malamas et al. reported the immunomodulation in tumor cells exposed to ^223^Ra and demonstrated that ^223^Ra enhanced T cell-mediated lysis of tumor cells by CD8+ T cells (82). In this study, breast (MDA-MB-231, ZR75-1), prostate (LNCaP, PC3), and lung (H1703, H441) carcinoma cells were first exposed for 96 h to ^223^Ra, delivering a radiation dose of 4 or 10 Gy. After establishing that there was no substantial tumor cell death, treated cells were co-cultured with CD8+ T cells specific for CEA, MUC, and brachyury epitopes. Their result showed a significant increase in T cell-mediated killing of all the cancer cells tested after 4 or 10 Gy of ^223^Ra. They concluded that sublethal doses of ^223^Ra enhance HLA-restricted, antigen-specific, CTL-mediated lysis of various human carcinomas and that such killing can be achieved by targeting a broad repertoire of tumor-associated antigens (82).

Leung et al. reported that ^223^RaCl_2_ decreased the splenic CD8+ T cells in Swiss mice (106). Their study aimed to determine the effect of ^223^RaCl_2_ on splenic immune cell population size and function. Three groups of mice received intravenously 0, 50, or 600 kBq/kg of ^223^RaCl_2_, and the spleens were harvested at days 5, 12, and 19 post-injections for analysis. Compared with the 0 kBq/kg group, there was no significant difference in the total number of harvested splenocytes in the 50 and 600 kBq/kg treated groups at all time points. However, treatment with 600 kBq/kg of ^223^RaCl_2_ was found to decrease splenic CD8+ T cells (*p* = 0.043) significantly and the 50 kBq/kg-treated group approached significance (*p* = 0.059) at day 19 post-treatment. In contrast, a very slight decrease in CD8+ T cells was observed before 19 days. Based on these results, they concluded that the effect of ^223^Ra on splenic cells is time- and dose-dependent.

Ferreira et al. observed a dose- and time-dependent decrease in the frequency of CD8+ T cells in the MyC-Cap TME of mice injected with 7.4 (∼300 kBq/kg) or 18.5 kBq (∼700 kBq/kg) of ^25^Ac-NM600, with the highest decrease in CD8+ T cells at 28 days post-injection with approximately 700 kBq/kg (69). They observed the highest decrease in CD8+ T cells at 19 days post-injection with 600 kBq/kg. However, no significant decrease in CD8+ T cells was observed in the Tramp-C1 TME after injection with 7.4 (∼300 kBq/kg) or 18.5 kBq (∼700 kBq/kg) of ^225^Ac-NM600 (105). Moreover, ^225^Ac-NM600 promoted a more active CD8+ T cell repertoire with increased expression of activation and proliferation markers such as CD44+, CD69+, and Ki67+ on CD8+ T cells, suggesting the induction of an immunogenic TME by ^225^Ac-NM600, which could be responsible for the anti-tumor response. However, no difference in tumor growth delay was observed after treatment with 18.5 kBq of ^225^Ac-NM600 + anti-CD8 antibody injected twice a week (for depletion of CD8 + cells). This indicated that the anti-tumor effect observed with ^225^Ac-NM600 did not depend on CD8+ T cell infiltration or stimulation but instead on Treg cell decrease in the TME (105).

##### 5.2.1.3 Natural killer (NK) cells

Leung et al., after investigating the effect of ^223^RaCl_2_ on splenic immune cell population size and function in Swiss Webster mice (106), reported a constant decrease in NK cell population at 5, 12, and 19 days post-injection of 600 kBq/kg of ^223^RaCl_2_, while 50 kBq/kg had no effect on NK cell population. As NK cells are a vital part of the immune cells to eliminate cancer cells, a decreased level of NK cells is counter-beneficial for anti-tumor immunity. Furthermore, the cytotoxic activity of NK cells in mice treated with 50 kBq/kg was significantly increased on day 12, while only a marginal increase in cytotoxicity was noted in the group treated with 600 kBq/kg. Based on these results, Leung et al. suggested that low activity of ^223^Ra would be beneficial to avoid a steady decrease in the NK cell population while potentiating their immune response (106).

##### 5.2.1.4 Dendritic cells

Gorin et al. studied the immunogenicity of bismuth-213 (^213^Bi) in mice with MC-38 adenocarcinoma using a vaccination approach (84). Mice were vaccinated 7 days before MC38 engraftment by subcutaneous injection of ^213^Bi-irradiated MC-38 cells (6-h incubation with 2.22 MBq/mL). Only 12% (3 of 25) of vaccinated mice developed tumors, compared to 84% (21 of 25) in the control group (non-vaccinated mice), suggesting that ^213^Bi-treated MC-38 cells are highly immunogenic and can elicit a robust anti-tumor response *in vivo*. Therefore, they explored *in vitro* the mechanisms supporting the α-particle-induced anti-tumor immune response by analyzing the immature bone marrow-derived dendritic cell (BMDC) phenotype after 48 h of incubation with conditioned medium from control or ^213^Bi-treated MC-38. Their results revealed that conditioned medium from ^213^Bi-treated MC-38 elicited a significant increase of 32% in CD40 expression, 44.8% in CD86 expression, and a non-significant upward trend of 4.4% in CD80 expression on BMDCs. The increased expression of these costimulatory molecules (CD40, CD80, and CD86 on the surface of BMDCs) is a characteristic of DC activation. However, no activation was observed when immature BMDCs were co-cultured with control media. These results suggest that ^213^Bi induces the release of soluble agents from MC-38 that are capable of activating DCs *in vitro*.

Furthermore, ^213^Bi increased the release of DAMPs such as HMGB1 and Hsp70 in the conditioned medium from treated MC-38 cells, which may contribute to the anti-tumor response by activating DCs (84). Similarly, Hagemann et al. demonstrated that the thorium-227 (^227^Th)-conjugate BAY2287411 targeting mesothelin induced an upregulation of DAMPs (83). After the exposure of OVCAR-3 cells to BAY2287411, upregulation of the DAMPs, calreticulin, HSP70, HSP90, and HMGB1 was observed. Another study also confirmed the upregulation of DAMPs after exposure of MC-38 hMSLN cells to thorium-227 delivered by a mesothelin-targeting compound, resulting in the subsequent activation of dendritic cells (107).

#### 5.2.2 Clinical studies

Only a few clinical studies have assessed the immunomodulatory effects of α-particle emitter-based RPT. Some are translational observational studies, and others investigate the clinical benefits of treatment combinations. Here, we will discuss the two primary studies that have evaluated the immunological changes in ^223^RaCl_2_-treated mCRPC patients by phenotyping the peripheral blood mononuclear cells (PBMCs) during ^223^Ra-treatment.

##### 5.2.2.1 CD8+ T cells

In a study reported in 2017, Kim et al. collected PBMCs before and 3–4 weeks after treatment with ^223^RaCl_2_ in 15 men with mCRPC and analyzed the CD8+ T cell population along with their subsets (naive, central memory, and effector memory) by flow cytometry (108). After IV administration of 50 kBq/kg, there was no change in the CD8+ T cell population. However, the proportion of effector memory CD8+ T cells expressing PD-1 was significantly reduced from 20.6% to 14.6% (108).

To better understand the immunological effects of ^223^Ra, Creemers et al. investigated the composition and abundance of circulating PBMCs in mCRPC patients before, during, and after treatment with ^223^RaCl_2_ (109). A total of 30 patients with mCRPC had their PBMCs collected, and longitudinal alterations in circulating immune cell populations were examined through immunophenotyping analysis. Patients received 6 monthly injections of 55 kBq/kg of ^223^RaCl_2_. They reported an increase in the proportion of CD8+ T cells expressing the immune checkpoint molecules PD-L1, ICOS, PD-1, or TIM-3, while the percentage of T cells within the PBMC population decreased during ^223^RaCl_2_ treatment. Their study was not limited to CD8+ T cells. They also reported a decrease in the total lymphocyte counts by approximately a factor of 2 during treatment, while monocyte counts remained relatively stable during this time. In addition to an increase in the proportion of T cells expressing inhibitory checkpoint molecules (PD-L1, PD-1, and TIM-3), an increase in the proportion of Treg and myeloid-derived suppressor cells (MDSC), two immunosuppressive subsets, was also observed during ^223^RaCl_2_ treatment. While no previous data on the effect of ^223^RaCl_2_ on Tregs or MDSCs are available, these findings are supported by several other studies reporting that ionizing radiation can lead to the accumulation of circulating and tumor-infiltrating Tregs (110–112) and MDSCs (113). A mechanistic understanding of the cause of this observation might be helpful to stimulate the immune system and optimize combined treatment approaches with RPT and immunotherapies.

α-particle emitters have diverse, profound, and unique immunostimulatory effects. The use of α-particle emitters as radiopharmaceuticals offers several advantages. α-particles have high linear energy transfer and a short path length, making them effective in killing cancer cells while minimizing damage to surrounding healthy tissue. α-particles may also be more effective in treating radioresistant tumors, providing an alternative option for patients who may not respond well to other forms of radiation therapy. This may be due to some of the immunomodulation occurring with the use of α-particle RPT. As interest increases in the field, it will be just as critical to determine the timing and dosing of α-particle RPT administration as it will be to investigate which immune populations are critical to achieving a complete response. Understanding the impact of α-RPT on immune cell populations is essential to elucidating the mechanisms behind its immunostimulatory effects. Such insights could pave the way for the development of combination therapies that act synergistically with α-RPT, potentially promoting tumor elimination and improving treatment outcomes for cancer patients.

#### 5.2.3 Toxicities induced by α-particle-emitter-based RPT

α-particle-emitting RPT can lead to hematologic toxicity, including anemia, lymphocytopenia, leukopenia, thrombocytopenia, and neutropenia. However, due to the shorter path length of α-particles, these toxicities tend to be less severe compared to those induced by β-particle emitters (115, 115). Table 3 summarizes the key hematological and non-hematological toxicities observed during the phase 3 trial evaluating the safety and efficacy of ^223^RaCl_2_ vs. placebo in patients with mCRPC and bone metastases.

**Table 3:** Summary of clinical toxicities of α-particle-emitter-based RPT (58).

Radionuclide	Radium-223			
Clinical indication	Metastatic prostate cancer			
Targeting agent	None			
Treatment arm	Placebo		Radium-223	
Patients (%)	*N* = 301 (100%)		*N* = 600 (100%)	
Adverse event	All grades	Grade ≥3	All grades	Grade ≥3
Hematologic				
• Anemia	92 (31)	40 (13)	187 (31)	76 (13)
• Thrombocytopenia	17 (6)	6 (2)	69 (12)	39 (6)
• Neutropenia	3 (1)	2 (1)	30 (5)	13 (3)
• Lymphopenia	NR	NR	NR	NR
Non-hematologic				
• Constipation	64 (21)	4 (1)	108 (18)	6 (1)
• Diarrhea	45 (15)	5 (2)	151 (25)	9 (2)
• Nausea	104 (35)	5 (2)	213 (36)	10 (2)
• Vomiting	41 (14)	7 (2)	111 (18)	10 (2)
• Fatigue	77 (26)	18 (6)	154 (26)	24 (5)

NR, not reported.

### 5.3 Effect of Auger electron-emitter-based RPT on immune cells

The probability of AE hitting immune cells present in the TME is very low because of their short range (< 1 µm). Even at the cellular level, AE ionization is so localized that AEEs must be delivered close to a sensitive cellular target (i.e., nuclear DNA, mitochondria, or cell membrane) to maximize their efficacy. For this reason, the immunomodulatory effect of AEE-RPT is the least explored among the forms of emitted radiation from RPT. Only two studies have investigated the effectiveness of AEE radioimmunoconjugates in eradicating the leukemic stem cell population in acute myeloid leukemia or in eliminating myeloid leukemia cells (116, 117). However, these studies are outside the scope of the present report. On the other hand, AEEs are also responsible for non-targeted bystander effects occurring in non-irradiated cells (57, 118). As more efforts are needed to elucidate the immune effects of AEE-based RPT, understanding the AE-induced bystander effects will be critical. This bystander effect involves communication between irradiated and non-irradiated cells through gap junctions or releasing cytokine signals to the extracellular matrix (119, 120). Signals mediated by the bystander effect include cell death, genomic instability, cell cycle, proliferation, and responses to radiation, including radionuclides (56). Both *in vitro* and *in vivo* models have been used to experimentally demonstrate the bystander effect with AEE. Using deoxyuridine labeled with the AEE ^125^I (^125^IUdR), Howell et al. labeled 50% and 10% of V79 cells, and after replating the cells for clonogenic survival, the observed cell viability in both groups was lower than predicted, implying the existence of intercellular communication between irradiated and non-irradiated cells (121). Using a xenograft model of human colon adenocarcinoma in nude mice and the AEE (^125^IUdR), Xue et al. demonstrated the bystander response in an *in vivo* model (122). While the bystander effect occurs with various radionuclides, it is crucial for the therapeutic effect of AEE due to their very short range in tissue. The bystander effect may also be mediated by exosomes, which have emerged as one of the most attractive and promising candidates to initiate bystander effects (123, 124). Double-stranded DNA contained in the exosomes is increasingly recognized for triggering immune responses by acting as DAMP signals (125–129). Taken together, these findings suggest the need for additional studies exploring AE-RPT-induced immune response.

#### 5.3.1 Toxicities induced by Auger electron-emitter-based RPT

AEEs are often considered to be less toxic compared to α- and β- emitters due to their short range in tissues, which limits their ability to traverse cellular membranes and cause widespread damage (130, 131). However, it is important to note that the emission of other radiation types, such as gamma rays or conversion electrons, in non-pure AEEs like ^111^In and ^195m^Pt could contribute to toxicity and should be investigated.

### 5.4 Combination of RPT with immune checkpoint inhibitors (ICI)

Given the abundance of preclinical evidence suggesting the immunostimulatory effects of RPT, the potential benefits of combining RPT with ICI was investigated. Whether using β- or α-particle-emitter-based-RPT in combination with ICI, preclinical studies have reported mixed results, with various studies reporting the therapeutic benefit of such combination therapy while others have reported no benefit over RPT or ICI monotherapy (133). While some of the results have been mixed, there has been a promising positive trend with these combinations. Several studies have shown the efficacy and safety of RPT and ICI combination therapy, along with publishing the immunostimulatory effects of the combinations (7, 8, 93). For example, a lower dose outside the typical therapeutic range of ^90^Y was used to improve tumor response, reduce spontaneously arising metastatic tumor burden, increase complete response rates, and prolong overall survival compared to ICIs or RPT alone (8). This study also focused on the inherent characteristics of ^90^Y for dosimetric guidance during treatment. Indeed, other groups have also begun to investigate the therapeutic benefits of combining RPT and ICI while simultaneously using RPT for imaging and dosimetry. Kleinendorst et al. suggested the incorporation of dosimetric tools to correlate the absorbed radiation dose with the immunological effects (133). Other parameters such as the characteristics of the TME, such as tumor metabolism, hypoxia, and immunogenicity, may impact the immunomodulatory effects of RPT agents and the response to ICI. Thus, a better understanding of these parameters in the context of a therapeutic combination with RPT will be critical to harnessing a potential clinical therapeutic benefit. The selection of the optimal pairing of RPT (β- or α-particle emitters) and ICI (PD-1/PD-L1 inhibitors or CTLA-4 inhibitors) will also be crucial in implementing an effective therapeutic combination, as several studies have shown the upregulation of immune susceptibility markers following RPT (7, 93). As with any combination therapy, determining the optimal timing of the combination will also be critical.

## 6 Conclusion

RPT is revolutionizing cancer management, especially for patients with metastatic disease. In addition to its direct cytotoxic effects on tumor viability through DNA damage, RPT has the ability to indirectly affect tumor burden by altering the immune system response. This modulation involves various mechanisms including, but not limited to, induction of ICD, alteration of the TME, and enhancement of antigen presentation. These immune-mediated effects contribute to a comprehensive response against cancer, highlighting the multifaceted nature of RPT's therapeutic impact. Understanding these dual mechanisms of action is essential for harnessing the full potential of RPT in cancer treatment strategies. In this review, we have covered several avenues that can be addressed, including a better understanding of the immune cells that play a role in the anti-tumor activity following RPT. Elucidating the mechanisms of RPT-mediated immune response, incorporating RPT dosimetry, selecting the optimal ICI and radionuclide for RPT, and determining the optimal timing, dose, and fractionation of RPT for the therapeutic combination with ICI will be critical to fully harnessing the therapeutic benefits of RPT while minimizing the toxicities, especially in patients with metastatic disease. Through dedicated exploration of these research paths, we can unveil the full therapeutic potential of RPT and pave the way for tailored, life-prolonging treatments for patients battling metastatic cancer. This proactive approach not only fosters a deeper understanding of RPT's mechanisms of action but also enables the development of novel strategies for optimizing treatment efficacy and minimizing adverse effects. Ultimately, these endeavors hold the promise of revolutionizing cancer care by offering personalized therapeutic solutions that address the unique needs of each patient.
